# Comparative Susceptibility of Sheep of Different Origins, Breeds and *PRNP* Genotypes to Challenge with Bovine Spongiform Encephalopathy and Scrapie

**DOI:** 10.1371/journal.pone.0143251

**Published:** 2015-11-20

**Authors:** Fiona Houston, Wilfred Goldmann, James Foster, Lorenzo González, Martin Jeffrey, Nora Hunter

**Affiliations:** 1 The Roslin Institute, University of Edinburgh, Easter Bush, Midlothian, United Kingdom; 2 Animal and Plant Health Agency (APHA–Lasswade), Pentlands Science Park, Bush Loan, Midlothian, United Kingdom; Creighton University, UNITED STATES

## Abstract

Sheep are natural hosts of the prion disease, scrapie. They are also susceptible to experimental challenge with various scrapie strains and with bovine spongiform encephalopathy (BSE), which affects cattle and has been accidentally transmitted to a range of other species, including man. Incidence and incubation period of clinical disease in sheep following inoculation is controlled by the *PRNP* gene, which has different alleles defined on the basis of polymorphisms, particularly at codons 136, 154 and 171, although other codons are associated with survival time, and the exact responses of the sheep may be influenced by other breed-related differences. Here we report the results of a long term single study of experimental scrapie and BSE susceptibility of sheep of Cheviot, Poll Dorset and Suffolk breeds, originating from New Zealand and of a wide range of susceptible and resistant *PRNP* genotypes. Responses were compared with those of sheep from a closed Cheviot flock of UK origin (Roslin Cheviot flock). The unusually long observation period (6–8 years for most, but up to 12 years for others) allows us to draw robust conclusions about rates of survival of animals previously regarded as resistant to infection, particularly *PRNP* heterozygotes, and is the most comprehensive such study reported to date. BSE inoculation by an intracerebral route produced disease in all genotype groups with differing incubation periods, although M112T and L141F polymorphisms seemed to give some protection. Scrapie isolate SSBP/1, which has the shortest incubation period in sheep with at least one VRQ *PRNP* allele, also produced disease following sub-cutaneous inoculation in ARQ/ARQ animals of New Zealand origin, but ARQ/ARQ sheep from the Roslin flock survived the challenge. Our results demonstrate that the links between *PRNP* genotype and clinical prion disease in sheep are much less secure than previously thought, and may break down when, for example, a different breed of sheep is moved into a new flock.

## Introduction

Classical scrapie in sheep is the prototype of the group of diseases known as transmissible spongiform encephalopathies (TSEs) or prion diseases, which include bovine spongiform encephalopathy (BSE) in cattle and Creutzfeld-Jakob disease (CJD) in man. These are fatal neurodegenerative disorders, characterised by long incubation periods (months to years) and typical histopathological changes (e.g. vacuolation, astrogliosis, neuronal loss). Studies in sheep and mice have revealed a strong genetic component to disease susceptibility, with survival time and incubation period principally under the control of a single gene, known as *PRNP*, which encodes PrP (prion protein), a normal host glycoprotein expressed widely in nervous and other tissues [1]. A key event in the pathogenesis of TSEs is the conformational alteration of the host PrP (termed cellular PrP, or PrP^C^) to a disease-associated form (termed PrP^Sc^), accumulation of which in the central nervous system, and often also in lymphoid tissue, is followed by the development of clinical signs[2].

Sheep are the natural hosts of a TSE (scrapie) which exists in classical and atypical forms and has different strains that are often experimentally transmissible. Amino acid polymorphisms of the sheep *PRNP* gene have been linked to susceptibility and resistance to natural and experimental infection with TSEs [1]. The most significant of these occur at codon 136, where the presence of valine (V) instead of alanine (A) results in greatly enhanced susceptibility to some strains of classical scrapie [3], and at codon 171, where the presence of arginine (R) instead of glutamine (Q) results in disease resistance [4]. Substitution of histidine (H) for arginine at codon 154 will lengthen survival and/or incubation periods in some cases [5]. A convenient short-hand for the different *PRNP* alleles gives the single letter code for the three amino acids at these positions e.g. ARQ. *PRNP* genetics is complex in sheep with many polymorphic codons, some of which have also been shown to influence disease susceptibility or incubation periods, such as the replacements of threonine for methionine at codon 112[6] and phenylalanine for leucine at codon 141 [7]. However, selective breeding programmes based on the three codon (136, 154, 171) genotype have been used successfully to reduce the incidence of classical scrapie at flock, breed and national levels by increasing the frequencies of resistant genotypes [8].

Animals with genotypes VRQ/VRQ and VRQ/ARQ are generally at highest risk of developing disease whereas classical scrapie disease in ARR/ARR sheep has only been reported in five animals out of thousands examined worldwide. PrP^Sc^ deposits in brain were found in all five but only two animals (one natural exposure, one after experimental challenge) were reported to have scrapie clinical signs [9, 10, 11]. ARQ/ARQ sheep are common in most breeds and represent a significant number of classical scrapie cases, but the representation of this genotype in scrapie cases varies in different countries and a proportion of ARQ/ARQ animals may survive scrapie in some outbreaks [1].

After more than a decade of breeding for resistance in Europe, high risk VRQ-carrying genotypes have been significantly diminished and ARR-carrying low risk genotypes represent now the majority, however AXQ/AXQ (X = R or H) animals still represent 10–30% of the populations [8]. As the *PRNP* selection criteria are entirely empirical and not based on a fundamental understanding of what causes disease resistance, the question remains whether further scrapie resistance breeding will be beneficial. The example of countries such as New Zealand (NZ) and Australia that have sheep populations which include scrapie-susceptible *PRNP* genotypes but remain entirely free of classical scrapie is likely the result of stringent disease security measures. However genes other than *PRNP* may also have a protective effect against scrapie and may have been selected in sheep breeds in NZ and Australia. In accordance with that observation, it has been claimed that polygenic variance was responsible for 21% of the total genetic variability related to susceptibility to scrapie in a Romanov flock in France, which points to genes other than *PRNP* with effects on susceptibility [12]. It is possible therefore that some of these non-*PRNP* genes may, in some scrapie outbreaks, override the *PRNP* effects and result in either the appearance of disease in animals thought to be relatively resistant, or lack of disease in susceptible animals. It is also possible that lengthening of incubation period, rather than full resistance, could create a reservoir in which slower less aggressive strains might adapt into new, faster and disease-causing strains with unknown host range.

This study therefore aimed to explore the extent of resistance and incubation period control in sheep using well characterised methods of experimental inoculation and as wide a range of susceptibility and resistance as possible. The outcome of experimental challenges of sheep with TSE agents is governed to a greater or lesser extent by factors such as the agent strain type and titre, *PRNP* genotype, non-*PRNP* genes, route of infection, sheep breed and age eg [13, 14, 15]. In this study we have used two TSE strains which target sheep of different *PRNP* genotypes: BSE (which has the widest known host range of any TSE strain) and SSBP/1 (which is a well characterised experimental scrapie source). BSE has shortest incubation periods in AXQ/AXQ sheep [1,2, 15,16] and SSBP/1 in contrast more efficiently targets animals encoding at least one VRQ allele [17, 18]. Representatives of two sheep populations, with different background and selection history, were inoculated–one from the Roslin Institute Cheviot flock (originating and maintained in the UK) and the other originating from New Zealand but also maintained in the UK. Although similar genotype/TSE strain challenges in sheep have been reported previously, overall numbers of animals studied are inevitably low due to the considerable cost of large animal studies and so this study adds to the accumulated data and information on disease susceptibility in sheep while exposing a wide range of different *PRNP* genotypes to TSE challenge in a single study. We assessed the capability of the agent to cause PrP^Sc^ accumulation in a particular genetic background, the degree of peripheral PrP^Sc^ deposition and the incubation period before clinical disease signs were observed. In non-clinical animals, which were observed for 6.5 years in most cases but up to 12 years in others, survival times with or without PrP^Sc^ deposition were also recorded. It is usual in sheep TSE studies to concentrate on the shorter incubation period models and fully susceptible genotypes but in this study we included sheep of *PRNP* genotypes expected to show little or no disease in response to challenge. In contrast to mouse models of TSE disease, sheep can be observed for much longer incubation and survival times after challenge due to their much longer lifespan. As a result these experimental models give a more accurate reflection of susceptibility/resistance to natural TSE strains limited only by the life expectancy of the species.

## Materials & Methods

### Sheep—origin, breeds and management

An outline of the experimental design is presented in Table 1. The New Zealand sheep were of Suffolk, Cheviot and Poll Dorset breeds which were either imported directly from New Zealand or derived from the breeding flock established from the imported sheep (the Defra scrapie-free flock) [18]. The sheep were housed throughout the study in a purpose-built experimental unit at the Institute for Animal Health (IAH), Compton, with strict procedures in place to minimize the risks of cross-contamination between groups, as previously described [17]. The whole project (including controls, described below) was reviewed and approved by the IAH (Compton) Animal Welfare and Ethics Committee, and the IAH (Edinburgh) Protocols and Ethics Committee and carried out under the authority of current UK Home Office licences. After challenge with BSE or scrapie, using anaesthesia and analgesics appropriate for the species, sheep were monitored daily, and once clinical signs consistent with TSE disease were confirmed, they were euthanized using anaesthetic overdose and according to UK Home Office approved procedures. Animals developing intercurrent illness were treated by veterinary surgeons and were euthanized as above if there was no response to treatment. Times between the dates of inoculation and death are, in this paper, presented as either incubation periods (IP) or survival time (ST) and defined as follows: incubation time for sheep positive for TSE clinical signs and positive for disease-related PrP (PrP^d^) detection; survival time for sheep which were negative for either or both of TSE clinical signs and PrP^d^. A number of animals survived without any clinical signs to the end of the experiment and were euthanized as above (between 2200 and 2500 days post-inoculation).

**Table 1:** Experimental design.

PRNP genotype	TSE challenge and route	Dose	Number of sheep challenged	Breed	Number of NZ sheep	Number of Roslin sheep
**VRQ/VRQ**	**Cattle BSE, intracerebral**	0.5ml, 10% brain homogenate	10	Cheviot	5	-
				Poll Dorset	5	-
**VRQ/ARQ**	ditto	ditto	10	Cheviot	5	-
				Poll Dorset	5	-
**VRQ/ARR**	ditto	ditto	11	Cheviot	5	2
				Poll Dorset	4	-
**ARQ/ARQ**	ditto	ditto	19	Cheviot	5	-
				Poll Dorset	4	-
				Suffolk	10	-
**ARQ/ARR**	ditto	ditto	22	Cheviot	5	2
				Poll Dorset	6	-
				Suffolk	9	-
**AHQ/ARR**	ditto	ditto	1	Cheviot	-	1
**ARR/ARR**	ditto	ditto	31	Cheviot	5	12
				Poll Dorset	4	-
				Suffolk	10	-
**VRQ/VRQ**	**SSBP/1, sub cutaneous**	2ml, 10% brain pool	18	Cheviot	5	8
				Poll Dorset	5	-
**VRQ/ARQ**	ditto	ditto	28	Cheviot	5	18
				Poll Dorset	5	-
**VRQ/AHQ**	ditto	ditto	7	Cheviot	-	7
**VRQ/ARR**	ditto	ditto	23	Cheviot	5	14
				Poll Dorset	4	-
**ARQ/ARQ**	ditto	ditto	22	Cheviot	4	3
				Poll Dorset	5	-
				Suffolk	10	-
**ARQ/AHQ**	ditto	ditto	1	Cheviot	1	7
**ARQ/ARR**	ditto	ditto	37	Cheviot	5	17
				Poll Dorset	5	-
				Suffolk	10	-
**AHQ/ARR**	ditto	ditto	3	Cheviot	-	3
**ARR/ARR**	ditto	ditto	27	Cheviot	6	6
				Poll Dorset	5	-
				Suffolk	10	-

Sheep used for comparison of response to TSE infection were Cheviots with the same range of *PRNP* genotypes from a well-studied flock previously known as the NPU Cheviots but now renamed as the Roslin Cheviot flock which were challenged using the same routes and equivalent doses of SSBP/1 and BSE as part of other studies [16,19]. These control animals are referred to as Roslin sheep in this paper.

Small parts of this study, with very limited *PRNP* genotype information, have been published previously [16,17,20] and this is indicated in the text where appropriate.

### *PRNP* genotyping

This study was set up based on *PRNP* genotypes at codons 136, 154 and 171, their polymorphisms being A136V, R154H and Q171R. At the end of the study three-codon genotypes of selected animals were confirmed by re-sequencing of the *PRNP* coding region which allowed us also to determine the genotypes for codons 112 and 141 with polymorphisms M112T and L141F. PCR amplification and sequencing were performed as described previously [15]. Based on these polymorphisms, six allelic variants were present in this study: M_112_V_136_L_141_R_154_Q_171_ (here: VRQ), MALHQ (here: AHQ), MALRR (here: ARR) MALRQ (here: ALRQ or ARQ), MAFRQ (here: AFRQ or ARQ), TALRQ (here: TARQ or ARQ).

### Experimental infection with TSEs, tissue collection and analysis

In breeds carrying the *PRNP* VRQ allele, such as Cheviot and Poll Dorset, scrapie occurs predominantly in VRQ/VRQ and VRQ/ARQ genotypes and is rare in ARQ/ARQ animals. Groups of five Cheviot and five Poll Dorset sheep with *PRNP* genotypes VRQ/VRQ, VRQ/ARQ, VRQ/ARR, ARQ/ARQ, ARQ/ARR and ARR/ARR were each challenged with BSE or scrapie. In Suffolk sheep, the VRQ allele is very rare, and natural scrapie occurs in the ARQ/ARQ genotype. In Suffolks, therefore ten sheep with *PRNP* genotypes ARQ/ARQ, ARQ/ARR and ARR/ARR were each challenged with BSE and scrapie. In some cases, group sizes were reduced by intercurrent deaths. Responses of the different breeds to TSE challenge were very similar so genotype groups were pooled to simplify data tables. In a few cases we have commented on a possible breed effect influencing results however these were not major and were the only notable instances throughout.

BSE challenges were by intracerebral inoculation of 0.5ml 10% BSE-infected cattle brain homogenate (i.e. equivalent to 0.05g BSE-infected brain), as previously described [16]. The BSE-infected cattle brains used for preparation of the inocula were obtained from the TSE Archive (now the APHA Biological Archive), and two different brains were used to prepare the inocula used for sheep challenges, hereafter referred to as BSE-I and BSE-II. Roslin sheep were inoculated with both BSE-I and BSE-II in different groups whereas New Zealand sheep all received BSE-II. Both BSE inocula were titrated by intracerebral inoculation in RIII mice and showed similar titres of 10^3.2^ ID_50_/g.

Scrapie challenges were by subcutaneous inoculation of 2ml 10% SSBP/1 sheep brain homogenate (i.e. equivalent to 0.2g scrapie-infected brain) [17], with New Zealand sheep being inoculated with the same batch of inoculum that had been used for previous challenges in the Roslin Flock animals. SSBP/1 transmits very poorly to RIII mice so cannot be titrated to compare directly with the BSE inoculum, however we have titrated SSBP/1 in tg338 mice at 10^7.4^ ID_50_/g. The titration figures for BSE and SSBP/1 in the two completely different mouse lines are both very high titre despite the apparent four-log difference as tg338 mice are much more sensitive to SSBP/1 than RIII mice are to BSE.

Following euthanasia, a detailed necropsy was carried out for every sheep in the study, during which a range of tissues was collected including brain, spleen, tonsil, mesenteric lymph node. Samples used in this study were fixed in neutral buffered formalin for histopathology or frozen at -80C for biochemical analysis or bioassay. Immunohistochemical (IHC) staining for PrP^d^ deposition in the different sheep tissues was carried out using antibodies BG4, R145 and/or P4 as previously described [21]. Detailed IHC analysis for this project is considerable and as such is outwith the scope of this genetics paper so will be published elsewhere. However details of the methods and scoring systems for positive staining of sheep brain and lymphoid tissues can be found in our previous publications for SSBP/1 [17] and BSE [22,23]. For the purposes of this paper, we have provided a simple positive/negative score in which detection of any disease-related PrP resulted in a positive score for the relevant animal.

## Results

### Association of *PRNP* genotype with sheep responses to intracerebral challenge with BSE

In New Zealand sheep, intracerebral inoculation of BSE-II inoculum resulted in infection and clinical disease in all six *PRNP* genotypes (based on codons 136, 154, 171), although with differing incubation periods and attack rates, as shown in Table 2. Two additional polymorphisms at codons 112 and 141 were identified after the experiment was initiated.

**Table 2:** Outcome of intracerebral challenges with cattle BSE in New Zealand sheep.

*PRNP* genotype	Codon 141 subgroup	Codon 112 subgroup	Number challenged	Clinically positive and IHC positive^a^		Clinically negative and IHC positive^a^		Clinically negative and IHC negative^a^	
				Number	Mean incubation period in days (±SD)	Number	Mean survival time post infection in days (±SD)	Number surviving <2000 days post infection (range of survival times)^b^	Number surviving >2000 days post infection (range of survival times in days)^c^
ARQ/ARQ	LL	MM	12	12	537 (±33)	0	NA	0	0
	LL	MT	1	0	NA	1	2414	0	0
	LL	TT	1	0	NA	0	NA	0	1 (2414)
	FF	MM	5	5	608 (±38)	0	NA	0	0
VRQ/VRQ	LL	MM	10	10	1099 (±44)	0	NA	0	0
VRQ/ARQ	LL	MM	7	7	875 (±77)	0	NA	0	0
	LF	MM	3	3	2017 (±56)	0	NA	0	0
ARR/ARR	LL	MM	19	10	1486 (±398)	0	NA	3 (822–1930)	6 (2224–2418)
VRQ/ARR	LL	MM	9	4	1846 (±72)	1	2228	2 (106,1906)	2 (2228,2360)
ARQ/ARR	LL	MM	15	4	2024 (±110)	4	2320 (±109)	3 (938–1744)	4 (2151–2312)
	LF	MM	5	0	NA	0	NA	3 (661–1428)	2 (2395,2396)

^a^ Immunohistochemistry results for PrP^d^ detection in brain sections.

^b^ These animals were culled before the end of the experiment due to intercurrent disease or welfare concerns.

^c^ These animals were culled at the end of the experiment.

NA not applicable

The shortest incubation periods were found in the ARQ/ARQ group of sheep (mean ± SD = 558 ± 47 days post infection) and the attack rate was 90%. The two sheep in this group that did not develop clinical signs were found to carry the methionine to threonine mutation at *PRNP* codon 112 (M112T); one was MARQ/TARQ and the other TARQ/TARQ. Although numbers are too small for statistical analysis, the survival of 2414 days post infection (dpi) for both of the T112 carriers makes them very different from the MARQ/MARQ sheep, which have a 100% attack rate and incubation periods of ~500–600 days. The MARQ/TARQ sheep was found to have weak staining for PrP^d^ in the brain when it was culled at 2414 dpi.

In ARQ/ARQ sheep homozygous for leucine at codon 141 (ALRQ/ALRQ: n = 12) the mean incubation period (537 ± 33 days) was shorter than in sheep homozygous for phenylalanine (AFRQ/AFRQ; n = 5) at 608 ± 38 days, and this difference was statistically significant (p<0.01). However, it should be noted that the 5 AFRQ/AFRQ sheep were of the Cheviot breed, while the ALRQ/ALRQ sheep were Poll Dorset and Suffolk. Among the latter, Poll Dorset sheep had the shortest incubation periods (data not shown).

Surprisingly, VRQ/VRQ sheep were also highly susceptible to BSE, with an attack rate of 100% and a mean incubation period of 1099 ± 44 days. The attack rate in VRQ/ARQ sheep was also 100%, but they fell into two distinct groups with regard to length of incubation period, according to the codon 141 amino acid on the ARQ allele. The first group of seven VLRQ/ALRQ sheep had incubation periods intermediate between those of ARQ and VRQ homozygotes (mean ± SD = 875 ± 77 days), and the remaining three VLRQ/AFRQ sheep had incubation periods that were more than 2.5 years longer (2017 ± 56 days; p<0.001).

Sheep carrying the ARR *PRNP* allele (homozygotes or heterozygotes) were susceptible to intracerebral challenge with BSE, but had lower attack rates and longer, more variable incubation periods than the other genotype groups. In the ARR/ARR group, 10 out of 19 sheep (52%) developed clinical signs, with incubation periods ranging from 1008 to 2299 days (mean ± SD = 1486 ± 398 days). Four out of nine VRQ/ARR sheep (44%) and four out of 20 ARQ/ARR sheep (20%) developed clinical signs with mean incubation periods of 1846 ± 72 days and 2024 ± 110 days, respectively. When the remaining sheep in these two groups were culled in the absence of clinical signs at >2000 dpi, 4 out of a total of 10 ARQ/ARR sheep and 1 out of a total of 3 VRQ/ARR sheep showed evidence of low level PrP^Sc^ accumulation in the brain.

Four sheep carrying the ARR allele (one each of ALRR/ALRR, ALRQ/ALRR, AFRQ/ALRR, and VLRQ/ALRR genotypes) were showing signs of ataxia (incoordination) and were originally scored as clinically positive, but TSE infection could not be confirmed by detection of PrP^d^ by IHC following necropsy. Brain homogenate from one of the AFRQ/ALRR animal, which developed ataxia and was culled at 1483 dpi, was inoculated into RIII mice, but there was no evidence of infectivity, as the mice did not develop clinical signs of TSEs and survived >600 days post infection. In contrast, brain homogenate from a positive control sheep (AFRQ/AFRQ, BSE incubation period 671dpi) produced the characteristic BSE incubation period (mean ± SD = 348 ± 22 dpi) and pathology in RIII mice (data not shown), and therefore it is likely that the four ataxic animals were clinically misclassified.

The results of two experiments involving intracerebral inoculation of Cheviot sheep from the Roslin Flock with cattle BSE are summarized in Table 3. Overall, the results from intracerebral BSE challenges in the RSF flock are broadly similar to those seen in New Zealand sheep in terms of incubation periods, but most of the group sizes are too small for comparison of attack rates. Interestingly, all of the ARR/ARR RSF Cheviot sheep (n = 6) inoculated with BSE-II succumbed to infection, although those inoculated with BSE-I had a lower attack rate (50%). The New Zealand ARR/ARR Cheviots (n = 5) had a 100% attack rate, while the attack rates in New Zealand ARR/ARR Poll Dorset and Suffolk sheep were lower (25% and 40% respectively), all with the same BSE-II inoculum. The reasons for the differences are not clear.

**Table 3:** Outcome of intracerebral challenges with cattle BSE in the Roslin Flock.

Cattle brain isolate	*PRNP* genotype^a^	Number challenged	Clinically positive and IHC positive^b^		Clinically negative and IHC negative^b^	
			Number	Incubation period in days (mean ±SD)	Number surviving <2000 days post infection (range of survival times in days)	Number surviving >2000 days post infection (range of survival times in days)
BSE-I	VRQ/ARR	2	1	1874	0	1 (2379)
	ARQ/ARR	2	2	1886, 1923	0	0
	AHQ/ARR	1	1	2353	0	0
	ARR/ARR^c^	6	3	1582 ±110	1 (1523^d^)	2 (2204 ^d^ ^,^ ^e^, 2242)
BSE-II	ARR/ARR^c^	6	6	1712 ±375	0	0

^a^ All animals had codon 141 LL genotype.

^b^ Immunohistochemistry results for PrPd detection in brain sections.

^c^ Previously published data.

^d^ Intercurrent deaths.

^e^ Tissues unavailable for analysis

### Association of *PRNP* genotype with incubation periods and attack rates following subcutaneous challenge with SSBP/1

The response to subcutaneous challenge with SSBP/1 in New Zealand sheep was very different to that observed following BSE challenge, as shown in Table 4. The shortest incubation periods and 100% attack rates were recorded for sheep carrying the VRQ allele (homozygous or heterozygous). For VRQ/VRQ sheep, the mean incubation period was 150 ± 15 days (n = 10), while for VRQ/ARR sheep it was 233 ± 38 days (n = 9). In VRQ/ARQ sheep, the incubation periods again appear to vary according to codon 141 genotype, with VLRQ/ALRQ animals having shorter incubation periods (mean ± SD = 192 ± 21 days; n = 6) than VLRQ/AFRQ animals (271 ± 37 days; n = 4). However, the VLRQ/ALRQ sheep were mostly Poll Dorsets, while the VLRQ/AFRQ sheep were Cheviots, and it is therefore difficult to distinguish the effect of 141 codon polymorphism from possible breed effects. The survival curves and overall incubation periods for the VRQ/ARQ and VRQ/ARR groups were very similar and in the latter, the shortest incubation periods were also found in Poll Dorsets (data not shown).

**Table 4:** Outcome of subcutaneous challenges with SSBP/1 in New Zealand sheep.

*PRNP* genotype	Codon 141 subgroup	Codon 112 subgroup	Number challenged	Clinically positive and IHC positive		Clinically negative and IHC negative	
				Number	Mean incubation period in days post infection (± SD)	Number surviving <2000 days post infection (range of survival times in days)^a^	Number surviving >2000 days post infection (range of survival times in days)^b^
VRQ/VRQ	LL	MM	10	10	150 ± 15	0	0
VRQ/ARQ	LL	MM	6	6	192 ± 21	0	0
	LF	MM	4	4	271 ± 37	0	0
VRQ/ARR	LL	MM	9	9	233 ± 38	0	0
ARQ/ARQ	LL	MM	13	12	1140 ± 226	1 (802)	0
	LL	MT	1	0	NA	0	1 (2780)
	FF	MM	4	4	1241 ± 53	0	0
	n.d.	n.d.	1	0	NA	1 (335)	0
ARQ/AHQ	LF	MM	1	0	NA	0	1 (2249)
ARQ/ARR	LL	MM	15	0	NA	3 (811–1730)	12 (2242–2779)
	LF	MM	4	0	NA	1 (518)	3 (2776–2777)
	n.d.	n.d.	1	0	NA	1 (222)	0
ARR/ARR	LL	MM	21	0	NA	4 (22–1729)	17 (2248–2779)

^a^ These animals were culled before the end of the experiment due to intercurrent disease or welfare concerns.

^b^ These animals were culled at the end of the experiment.

NA, not applicable

n.d., not determined

New Zealand sheep with *PRNP* genotype ARQ/ARQ were also susceptible to SSBP/1, although they had much longer and more variable incubation periods than those observed in animals carrying the VRQ allele. In the ALRQ/ALRQ (n = 14) subgroup, all except one animal developed clinical signs of scrapie and were confirmed positive by IHC, giving an attack rate of 86% but with very variable incubation periods, ranging from 878 to 1526 days (mean ± SD = 1140 ± 226 days). In the AFRQ/AFRQ subgroup (n = 4) sheep (all Cheviots), incubation periods were more consistent (mean ± SD = 1241 ± 53 days). Three out of nineteen sheep in the ARQ/ARQ challenge group were clinically negative, and PrP^d^ staining was not detected in any of the tissues (brain or lymphoid) examined. Two of these negative sheep were culled because of intercurrent illness at 335 days (codon 112 and 141 subtypes not determined) and 802 days (112MM, 141LL) post infection, respectively. The third sheep remained healthy until the end of the experiment (2780 days), and was 112MT and 141LL. An additional single sheep originally assigned to the ARQ/ARQ challenge group was found when genotypes were confirmed at the end of the study to be AFRQ/ALHQ. This animal also survived until the end of the experiment (2249 days).

In contrast to the results of the intracerebral BSE challenge, New Zealand sheep with ARQ/ARR and ARR/ARR genotypes appeared completely resistant to sub-cutaneous infection with SSBP/1. There were no clinical signs of scrapie recorded in either group and IHC performed on brain and lymphoid tissues from all sheep that survived longer than 2000 days (15 ARQ/ARR and 17 ARR/ARR) failed to find any evidence of subclinical infection.

The results of subcutaneous challenges with SSBP/1 in the Roslin Flock are summarized in Table 5. As observed in the New Zealand sheep, animals carrying the VRQ *PRNP* allele were fully susceptible to infection, with 100% attack rates, while ARQ/AHQ, ARQ/ARR, AHQ/ARR and ARR/ARR animals were resistant. However, in contrast to the New Zealand sheep, Roslin sheep with ARQ/ARQ genotypes were also resistant to infection with SSBP/1, as are Roslin ARQ/AHQ genotypes.

**Table 5:** Outcome of subcutaneous challenges with SSBP/1 in the Roslin Flock.

*PRNP* genotype	Codon 141 subgroup	Number challenged	Clinically positive and IHC positive		Clinically negative and IHC negative	
			Number	Mean incubation period in days post infection (± SD)	Number surviving <2000 days post infection (range of survival times in days)^a^	Number surviving >2000 days post infection (range of survival times in days)^b^
VRQ/VRQ	LL	8	8	170 (± 27)	0	0
VRQ/ARQ	n.d.	10	10	273 (± 37)	0	0
	LF	6	6	230 (± 40)	0	0
	LL	2	2	291, 351	0	0
VRQ/AHQ	LL	7	7	361 (± 65)	0	0
VRQ/ARR	LL	14	14	323 (± 34)	0	0
ARQ/ARQ	n.d.	3	0	NA	1 (1860)	2 (2331, 2532)
ARQ/AHQ	LL/n.d.	7	0	NA	3 (1242–1994)	4 (2284–2813)
ARQ/ARR	LL/LF/n.d.	17	0	NA	7 (567–1601)	10 (2107–4256)
AHQ/ARR	LL	3	0	NA	1 (1693)	2 (2284,2813)
ARR/ARR	LL	6	0	NA	1 (568)	5 (2284–2937)

^a^ These animals were culled before the end of the experiment due to intercurrent disease or welfare concerns.

^b^ These animals were culled at the end of the experiment.

NA, not applicable

n.d., not determined

There were also differences in the mean incubation periods recorded for the Roslin and New Zealand sheep groups carrying the VRQ allele, which became more pronounced when New Zealand Cheviot and Poll Dorset sheep were compared separately. When the mean incubation periods for VRQ/VRQ, VRQ/ALRQ and VRQ/ARR are compared between the Cheviots from both the Roslin Flock and New Zealand sheep, the latter had on average 17% shorter incubation periods. More surprising is the difference in the increase of the mean incubation period from a VRQ/VRQ to VRQ/ARR, in other words the resistance effect of the ARR allele. Whereas in the Roslin Cheviots the increase was 190%, in NZ Cheviots this increase was only 77% and in the Poll Dorset it was only 28%. As the AHQ allele is relatively common in the Roslin sheep, an additional group of sheep with the VRQ/AHQ genotype were challenged in these experiments, and had incubation periods comparable to those recorded for VRQ/ARR sheep (361 ± 65 days; n = 7).

### Association of *PRNP* genotype with PrP^d^ deposition in lymphoid tissues of NZ sheep

**Fig 1:**
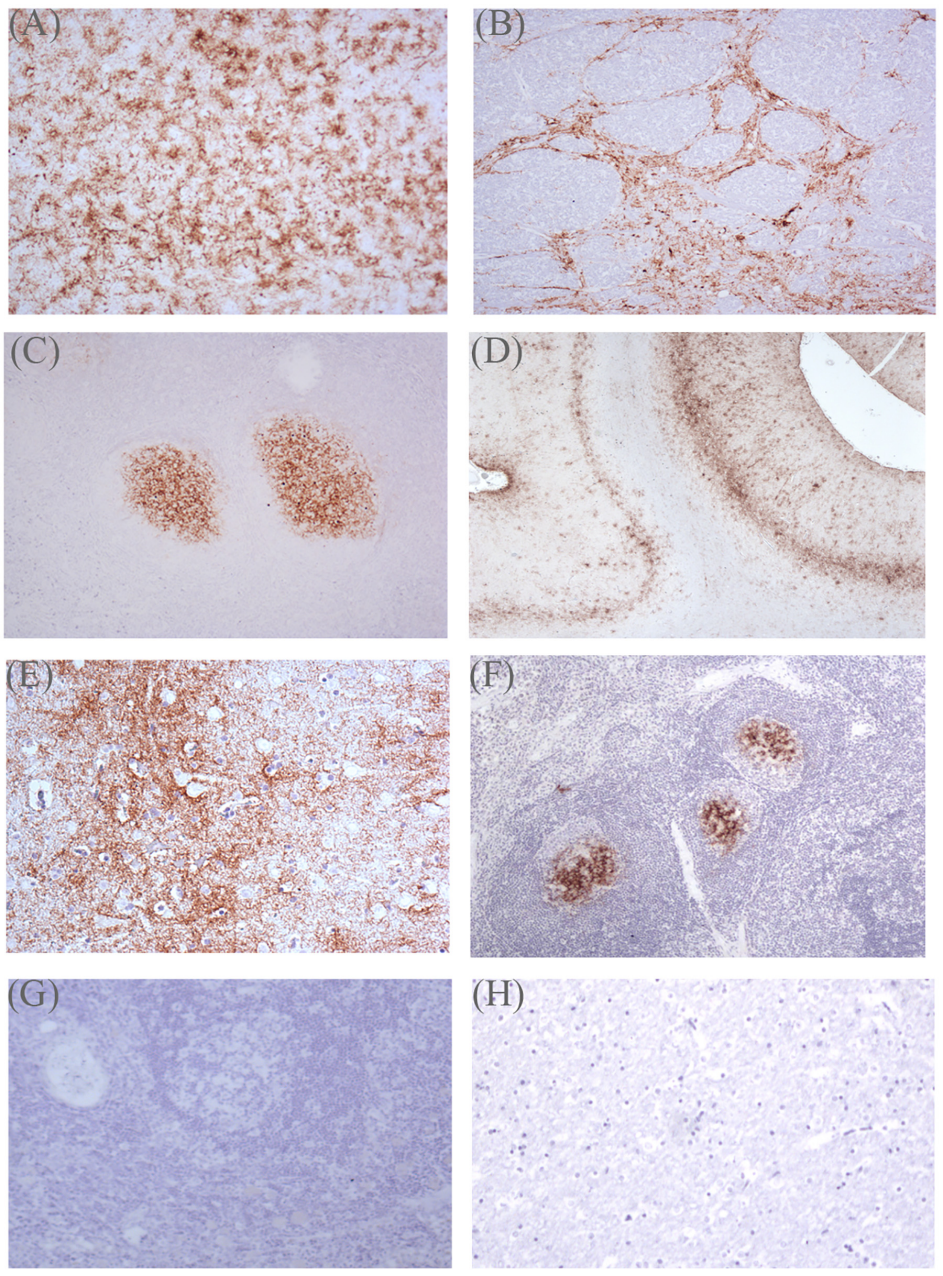
Detection of disease-related PrP^Sc^ in IHC of brain and lymphoid tissues of TSE inoculated sheep. IHC carried out using anti-PrP antibody BG4. (A), (B) and (C) positive staining in tissues from three sheep clinically affected following intracerebral inoculation with BSE. (A) ALRQ/ALRQ thalamus (x10); (B) AFRQ/AFRQ medulla (x10); (C) ALRQ/ALRQ mesenteric lymph node (x10). (D), (E) and (F) positive staining in tissues from three sheep clinically affected following sub-cutaneous inoculation with SSBP/1 scrapie. (D) VLRQ/VLRQ frontal cortex (x4); (E) VLRQ/ALRQ basal ganglia (x20); (F) VLRQ/AFRQ prescapular lymph node. (G) negative staining in tonsil of non-clinical AFRQ/ALRR BSE inoculated sheep. (H) negative staining in basal ganglia of non-clinical SSBP/1 ALRR/ALRR inoculated sheep.

A number of lymphoid tissues were collected from each sheep at post mortem, and for a proportion of animals from each genotype group these tissues were examined by IHC to determine the extent of PrP^d^ deposition. The details of the IHC will be published elsewhere. However the simple positive/negative results for both intracerebral BSE- and sub-cutaneous SSBP/1-challenged New Zealand sheep are summarized in Table 6, along with the corresponding IHC results for brain sections. Examples of positive and negative staining in brain and lymphoid tissues, are illustrated in Fig 1. Among the animals for which both brain and lymphoid tissues were examined, there were none that showed PrP^d^ deposition in lymphoid tissues alone i.e. in the absence of positive staining in the brain. In BSE-challenged sheep, out of eight clinically affected ARQ/ARQ sheep (both 141LL and 141FF) examined, all had PrP^d^ deposits in the three lymphoid tissues examined (spleen, mesenteric lymph node, tonsil). Sheep of other *PRNP* genotypes showed more restricted distribution of PrP^d^ in lymphoid tissues following BSE infection. As Table 6 shows, between 50% and 70% of the tested lymphoid tissues from VRQ/VRQ sheep were positive however, as not all sheep were the same, this actually equated to nine out of ten animals having at least one positive lymphoid tissue. Similarly with VRQ/ARQ sheep, four out of ten animals had at least one positive lymphoid tissue. There was no evidence of PrP^d^ deposition in lymphoid tissues from clinically or sub-clinically BSE-infected sheep in the VRQ/ARR, ARQ/ARR or ARR/ARR groups apart from one single sheep of ARQ/ARR genotype which was positive in spleen. In SSBP/1-challenged sheep, VRQ/VRQ sheep showed consistent PrP^d^ deposition in all lymphoid tissues examined, while VRQ/ARR sheep had a much more restricted distribution, with six out of nine infected sheep showing positive results in usually only one lymphoid tissue (most commonly tonsil). In SSBP/1-infected VRQ/ARQ and ARQ/ARQ sheep, PrP^d^ was also detected in almost all lymphoid tissues examined, with only one animal in each group (VLRQ/AFRQ and ALRQ/ALRQ respectively) negative in one of the three tissues.

**Table 6:** The distribution of PrP^d^ in lymphoid tissues of BSE- and SSBP/1-infected New Zealand sheep.

*PRNP* genotype*	No. clinical cases/no. inoculated with BSE (intracerebral)	BSE: no. positive tissues/no. tissues examined				No. clinical cases/no. inoculated with SSBP/1 (sub- cutaneous)	SSBP/1: no. positive tissues/no. tissues examined			
		Brain	Tonsil	MLN	Spleen		Brain	Tonsil	MLN	Spleen
VLRQ/VLRQ	10/10	10/10	6/10	5/10	7/10	10/10	10/10	10/10	10/10	10/10
VLRQ/ALRQ	7/7	7/7	1/7	1/6	1/7	6/6	6/6	6/6	6/6	6/6
VLRQ/AFRQ	3/3	3/3	1/3	1/3	1/3	4/4	4/4	4/4	3/4	3/4
ALRQ/ALRQ	12/12	12/12	4/4	4/4	4/4	12/14	12/14	11/14	11/13	12/14
AFRQ/AFRQ	5/5	5/5	4/4	4/4	4/4	4/4	4/4	4/4	4/4	4/4
VLRQ/ALRR	4/9	5/9	0/7	0/7	0/7	9/9	9/9	3/9	1/9	2/9
ALRQ/ALRR	4/15	8/15	0/10	0/12	0/12	0/15	0/15	0/15	0/15	0/15
AFRQ/ALRR	0/5	0/5	0/1	0/1	0/1	0/4	0/4	0/4	0/4	0/4
ALRR/ALRR	10/19	10/19	0/14	0/14	0/14	0/20	0/20	0/20	0/20	0/20

* The results for animals that had polymorphisms at codons 112 (112MT and 112TT) or 154 (154RH) were excluded from this table as none had positive staining in lymphoid tissues.

MLN = mesenteric lymph node

## Discussion

The outcome of experimental TSE infection in a permissive host (e.g. sheep, mice), in terms of attack rate (defined in this study as the proportion of inoculated individuals that developed clinical signs confirmed by detection of PrP^Sc^ post mortem), incubation period and neuroanatomical distribution of brain lesions, is influenced by many different factors. The best understood of these include age at inoculation and *PRNP* genotype, as well as the dominant strain of agent within the inoculum, and experimental factors such as the route of inoculation and infectious dose administered. The results of our experiment illustrate this, as inoculating groups containing two very different populations of Cheviots of similar age, breed and *PRNP* genotype with two TSE sources (BSE and SSBP/1) by different routes produced markedly different outcomes, almost certainly related to the source, or strain, of the inoculum.

In this, and previous studies, we have used immunohistochemical detection of disease-related PrP as a means to confirm, or rule out, that an animal was clinically affected by scrapie or BSE and it should be borne in mind that our negative animals could be subclinically infected and harbour levels of prion protein below the limits of the method to detect. However our results do provide information allowing comparison of the relative susceptibility of sheep of different *PRNP* genotypes to infection. In a previous study [24], the shortest incubation periods following experimental infection of sheep with scrapie were recorded when the *PRNP* genotype of the source inoculum was matched to that of the sheep being challenged (e.g. when VRQ/VRQ sheep were inoculated with brain homogenate from VRQ/VRQ, rather than ARQ/ARQ, scrapie cases), and similar results have been reported by others [25]. Similarly, in our experiments, when New Zealand sheep were inoculated with SSBP/1 inoculum (derived from scrapie-affected sheep carrying the VRQ allele), the shortest incubation periods were found in sheep with VRQ/VRQ, VRQ/ARQ and VRQ/ARR genotypes, and incubation periods in ARQ/ARQ sheep were much longer. On the other hand, in New Zealand sheep inoculated with BSE inoculum (derived from cattle brains, in which the *PRNP* sequence is closest to the sheep ARQ allele) the shortest incubation periods were recorded in ARQ/ARQ genotypes, with longer incubation periods in sheep carrying the VRQ allele. In terms of the prion hypothesis, strain characteristics have been said to be related to the dominant conformation adopted by the misfolded PrP^Sc^ protein from a range of possible conformers [26]. In this hypothesis, maintenance and propagation of the dominant conformer on sub-passage in another host is favoured in part by matching of the PrP primary protein sequence in donor and recipient; where there is a mismatch, another conformer present at lower levels may be selected, resulting in longer incubation periods and/or incomplete attack rates, and sometimes a change in strain characteristics [27].

The other interesting difference between the BSE and scrapie inocula is the response of sheep that carry the ARR *PRNP* allele as homozygous or heterozygous genotypes. These are considered to be highly resistant to classical scrapie because of their under-representation among natural scrapie cases [1]. Breeding strategies to reduce the incidence of scrapie have resulted in larger numbers of such sheep[8] however they are known to be affected by atypical scrapie [28] and are clearly not incapable of supporting a TSE infection. In our BSE-inoculated ARR/ARR New Zealand sheep, 52% of animals developed clinical signs (with longer and much more variable incubation periods than those of ARQ/ARQ and VRQ/VRQ genotypes), and had detectable PrP^d^ deposition in the brain, but not lymphoid tissues following necropsy. In contrast, none of the ARR/ARR New Zealand sheep inoculated with SSBP/1 showed clinical signs or evidence of PrP^d^ deposition in brain and lymphoid tissues. Similarly, five ARR/ARR Sarda sheep inoculated intracerebrally with cattle BSE developed clinical signs with incubation periods ranging from 1495 to 1751 days post infection [29] and ARR/ARR sheep inoculated with cattle BSE by peripheral routes (oral, intrasplenic, intraperitoneal) appeared to sustain the infection, although none developed clinical disease [30, 31]. One reason for the difference in results between the responses of ARR/ARR animals to BSE and scrapie animals could be the route of infection, since intracerebral inoculation of TSE agents is generally regarded as the most efficient method of transmission. However, intracerebral challenges of ARR/ARR sheep with scrapie from ARQ/ARQ or VRQ/VRQ sources have been negative [1, 16, 32] or at best highly inefficient [11]. Taking these results together, it would appear that ARR/ARR genotype sheep are unusually susceptible to BSE infection compared to common classical scrapie isolates and should BSE ever recur these sheep might be vulnerable to infection.

When our New Zealand sheep heterozygous for the ARR *PRNP* allele were inoculated with SSBP/1, ARQ/ARR animals appeared to be totally resistant to infection, while in VRQ/ARR sheep it produced a 100% attack rate, with incubation periods that were not significantly different from VRQ/ARQ sheep. These results agree with those obtained from previous subcutaneous challenges of Roslin Cheviot sheep with SSBP/1 (Table 5). In another study of oral challenges of sheep with scrapie there were similar findings, with incubation periods lengthening in the order VRQ/VRQ (198 ± 4 days) < VRQ/ARQ < VRQ/ARR < ARQ/ARQ (540 ± 3 days) [33]. However, that study also found that ARQ/ARR sheep were partially susceptible to this challenge, with a 64% attack rate and a mean incubation period of 2252 days (± 122 days SD). However the latter study used an inoculum made from pooled brains of 17 sheep of five different breeds and a range of *PRNP* genotypes, including ARQ/ARQ, making it more likely that it contained a broader range of strains than SSBP/1, which was isolated from, and has been repeatedly passaged in, sheep carrying the VRQ *PRNP* allele. In addition, the oral scrapie challenges [33] were performed in lambs less than three weeks old, when susceptibility to oral TSE infection is significantly greater than in older animals, such as those used in our experiments [15].

Natural scrapie has only rarely been reported in VRQ/ARR sheep and the reasons for the high attack rate and relatively short incubation periods in VRQ/ARR sheep infected experimentally are unclear. However it is possible that natural infection mechanisms for the initial uptake and/or replication of the scrapie agent from another animal or contaminated environment are relatively inefficient in VRQ/ARR sheep, and that experimental infection with brain homogenate (from VRQ-carrying scrapie cases) is able to by-pass this barrier in some way. The results may also be dose-related as experimental inoculation would be expected to deliver a large dose of infection compared to that picked up naturally.

In BSE-inoculated New Zealand sheep, a proportion of animals in the VRQ/ARR (44%) and ARQ/ARR (20%) *PRNP* genotypes groups also developed clinical disease. Although there was a clear tendency towards longer incubation periods in the ARR heterozygous animals compared to homozygotes, the great variability in the latter group resulted in a certain amount of overlap. However a number of infected sheep (PrP^d^ positive) were identified among ARR heterozygotes culled at the end of the experiment suggesting that clinical disease might have developed should the animals have lived longer. These observations are similar to the observation of over-dominance of *PRNP* alleles in mice challenged by certain scrapie strains[34] and are consistent with the hypothesis that the two *PRNP* alleles do not act independently in the control of pathogenesis in the heterozygote sheep.

Another possible example of over-dominance in our experiments was associated with heterozygosity at *PRNP* codon 141 in VRQ/ARQ sheep. While VLRQ/ALRQ sheep inoculated with BSE had incubation periods intermediate between those of ALRQ/ALRQ or AFRQ/AFRQ and VLRQ/VLRQ sheep, the very long incubation periods in VLRQ/AFRQ sheep were similar to those seen in ARR heterozygous animals. As in mice, this effect was strain dependent, since codon 141 heterozygosity was not associated with a similar dramatic effect on incubation periods in VRQ/ARQ sheep inoculated with SSBP/1. Although the incubation periods in VLRQ/AFRQ sheep inoculated with SSBP/1 were on average about 80 days longer than in VLRQ/ALRQ sheep, and the difference was statistically significant, this could simply be a breed difference since all the sheep carrying the 141F polymorphism were Cheviots. We noted very few other effects which could be related to breed. As we have reported previously in different studies [15, 35], AFRQ/AFRQ sheep intracerebrally inoculated with BSE had longer incubation periods (608 ± 38 days) than ALRQ/ALRQ sheep (537 ± 33 days). In sheep inoculated with SSBP/1, incubation periods were also longer on average in AFRQ/AFRQ sheep compared to ALRQ/ALRQ sheep, but the difference was not statistically significant because of the very large variability of incubation periods in the latter group. These results are similar to those of others[24], who found that the 141F polymorphism was associated with prolonged incubation periods in ALRQ/AFRQ, but not in VLRQ/AFRQ, sheep challenged orally or subcutaneously with two sources of classical scrapie.

Three of the New Zealand sheep used in these experiments were also found to carry the M → T polymorphism at codon 112; two of these (MARQ/TARQ and TARQ/TARQ, respectively) were challenged with BSE, and the other (MARQ/TARQ) with SSBP/1. The polymorphism appeared to have a protective effect, with all three sheep remaining healthy for prolonged periods (3–5 years) after the last similarly challenged MARQ/MARQ sheep had developed clinical signs and been culled. However, weak positive staining for PrP^d^ was found in the brain, but not lymphoid tissues, of one of the BSE-challenged sheep (112MT) after post mortem, indicating that it may have been infected. The protective effect of the T112 polymorphism has been previously reported in sheep experimentally infected with scrapie and BSE[24, 36,37, 38, 39], although in scrapie-infected 112MT sheep it appeared to result in prolonged incubation periods rather than complete protection from infection.

Comparison of the results of SSBP/1 and BSE challenges in New Zealand sheep with previous similar challenges of Roslin sheep did not reveal any major differences in susceptibility, apart from in the ARQ/ARQ *PRNP* genotype when inoculated with SSBP/1. While all New Zealand sheep with this genotype (apart from one animal which carried the M112T polymorphism) became infected and developed clinical disease, three ARQ/ARQ Roslin sheep showed no evidence of infection when culled at time points after infection that were at least 334 days beyond the longest incubation period recorded in their New Zealand counterparts. This result suggests that, although polymorphisms within the protein-coding region of the *PRNP* gene may be the most important factor that determines susceptibility to TSE infection and disease, there are additional host genetic factors that modulate the response. These could arise from polymorphisms within non-coding regions of the *PRNP* gene, or in other parts of the genome. In cattle, an insertion/deletion polymorphism within the promoter region of the *PRNP* gene has been associated with susceptibility to BSE [40] and polymorphisms in promoter and 3’ untranslated region (UTR) of *PRNP* in sheep have been predicted to have effects on PrP^C^ expression [39, 41].

In this study we have confirmed the complex links and associations between *PRNP* genotype and TSE susceptibility in sheep and extended the understanding of TSE strain/genotype/breed combinations. Although general rules have been, and continue to be, applied to sheep breeding in order to reduce incidence of scrapie, it can be seen clearly that these rules are less secure than has previously been believed, especially if sheep, particularly with heterozygote *PRNP* genotypes, are exposed to novel TSE strains or if different breeds of animals are brought into a flock.
